# Overexpression of Mcl-1 confers resistance to BRAF^V600E^ inhibitors alone and in combination with MEK1/2 inhibitors in melanoma

**DOI:** 10.18632/oncotarget.5755

**Published:** 2015-10-14

**Authors:** Neel M. Fofaria, Dennie T. Frederick, Ryan J. Sullivan, Keith T. Flaherty, Sanjay K. Srivastava

**Affiliations:** ^1^ Department of Biomedical Sciences and Cancer Biology Center, Texas Tech University Health Sciences Center, Amarillo, TX, USA; ^2^ Harvard Medical School, Boston, Massachusetts, USA; ^3^ Division of Medical Oncology, Massachusetts General Hospital Cancer Center, Boston, Massachusetts, USA

**Keywords:** BRAF inhibitors, de novo and acquired resistance, MEK1/2 inhibitors, Mcl-1, combination therapy

## Abstract

Melanoma harboring BRAF mutations frequently develop resistance to BRAF inhibitors, limiting the impact of treatment. Here, we establish a mechanism of resistance and subsequently identified a suitable drug combination to overcome the resistance. Single treatment of BRAF mutant melanoma cell lines with vemurafenib or dabrafenib (BRAF inhibitors) alone or in combination with trametinib (MEK1/2 inhibitor) resulted in overexpression of Mcl-1. Overexpression of Mcl-1 in A375 and SK-MEL-28 by transfection completely blocked BRAF and MEK1/2 inhibitor-mediated inhibition of cell survival and apoptosis. Melanoma cells resistant to BRAF inhibitors showed massive expression of Mcl-1 as compared to respective sensitive cell lines. Silencing of Mcl-1 using siRNA completely sensitized resistant melanoma cells to growth suppression and induction of apoptosis by BRAF inhibitors. *In vivo*, vemurafenib resistant A375 xenografts implanted in athymic nude mice showed substantial tumor growth inhibition when treated with a combination of vemurafenib and Mcl-1 inhibitor or siRNA. Immunohistochemistry and western blot analyses demonstrated enhanced expression of Mcl-1 and activation of ERK1/2 in vemurafenib-resistant tumors whereas level of Mcl-1 or p-ERK1/2 was diminished in the tumors of mice treated with either of the combination. Biopsied tumors from the patients treated with or resistant to BRAF inhibitors revealed overexpression of Mcl-1. These results suggest that the combination of BRAF inhibitors with Mcl-1 inhibitor may have therapeutic advantage to melanoma patients with acquired resistance to BRAF inhibitors alone or in combination with MEK1/2 inhibitors.

## INTRODUCTION

Melanoma, a malignant transformation of melanocytes accounts for the highest number of skin cancer related deaths with a 5-year survival probability of less than 5% [1]. BRAF mutation is observed in almost 60% melanomas [2, 3]. Most common mutation in BRAF is a single substitution of valine to glutamic acid at codon 600 (V600E), accounting for almost 90% BRAF mutations in melanoma [3–5]. Selective inhibitors targeting BRAF^V600E^ have shown significant clinical activity in the patients with late stage metastatic melanoma amongst which vemurafenib has been recently approved by US-FDA [6–8]. In spite of promising initial response, there have been several recent reports of acquired resistance within 6–9 months of treatment with BRAF inhibitors in most of the patients [8, 9]. Moreover, about 20–30% patients develop squamous cell carcinoma and more develop tumor recurrence limiting vemurafenib therapy as well as other BRAF targeted therapies [10, 11]. The ‘acquired resistance’ occurring in the tumors that were earlier sensitive to BRAF inhibitor treatment has emerged as a major obstacle in the treatment of the patients with late stage metastatic melanoma with BRAF^V600E^ mutation leading to poor prognosis. Therefore, a combination of BRAF inhibitor (dabrafenib) and MEK1/2 inhibitor (trametinib) was approved in early 2014 for the treatment of metastatic melanoma, to which incidences of resistance has already been observed [12, 13]. Hence, identification of the mechanism behind the resistance and formulating a drug combination to overcome resistance is of prime importance.

Myeloid cell leukemia – 1 (Mcl-1) is pro-survival member of Bcl-2, which is known to promote oncogenesis not through cell proliferation but by inhibition of apoptosis, hence leading to immortalization of malignant cells [14, 15]. Its expression is regulated by transcription factors like STATs, cAMP response elements and NFκB [16]. Mcl-1 is frequently overexpressed in a variety of human cancers thereby providing protection to the tumor cells from apoptosis [17, 18]. Hence, Mcl-1 has been identified as an important target in majority of human cancers [19, 20] and several therapeutic strategies focusing on Mcl-1 inhibition are currently under development [21–25].

In this study, we have established that resistance to BRAF inhibitors such as vemurafenib or dabrafenib alone or in combination with MEK1/2 inhibitor (trametinib) in melanoma cells was due to overexpression of Mcl-1. Moreover, our results suggest that combination of BRAF inhibitors with Mcl-1 targeted therapies were successful in overcoming acquired resistance in melanoma *in vitro* and *in vivo*.

## RESULTS

### Vemurafenib treatment induces Mcl-1 expression in melanoma cells

**Figure 1:**
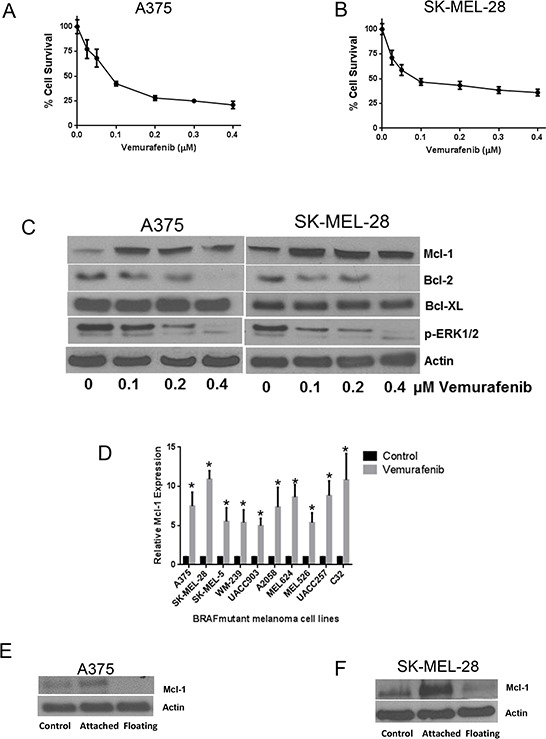

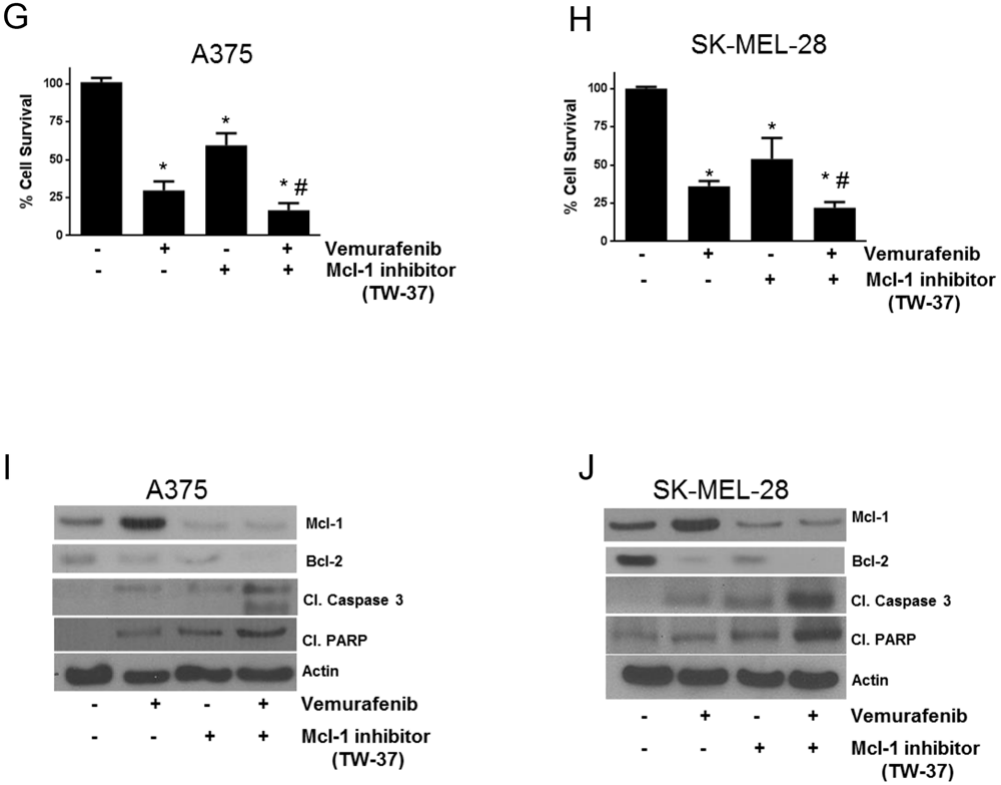
Vemurafenib treatment induces Mcl-1 expression in melanoma cells **A.** A375 and **B.** SK-MEL-28 cells were treated with various concentrations of vemurafenib for 72 hours. Following the treatment, cells were stained with Sulforhodamine B and the surviving cells were quantitated spectrophotometrically. The experiment was performed at least three times independently, each time with eight replicates and the data expressed as mean ± S.D. **C.** A375 and SK-MEL-28 cells were treated with 0.1, 0.2 and 0.4 μM vemurafenib for 72 hours. Following treatment, cell lysates were subjected to western blotting and analyzed for Mcl-1 expression. Each experiment was performed at least three times independently. **D.** Ten BRAF mutant cell lines were treated with 0.4 μM vemurafenib for 72 hours. The protein was collected, subjected to western blotting and analyzed for Mcl-1 expression. Fold increase in Mcl-1 expression was calculated by densitometric analysis. Actin was used as a loading control. Each experiment was performed at least three times independently. **E.** A375 and **F.** SK-MEL-28 cells were treated with 0.4 μM vemurafenib for 72 hours. Following the treatment, live cells were separated from dead floating cells and the lysates were prepared. The protein was subjected to western blotting and analyzed for Mcl-1 expression. Each experiment was performed three times independently. TW-37 enhanced the efficacy of vemurafenib in melanoma cells by inhibition of Mcl-1. **G.** A375 and **H.** SK-MEL-28 cells were treated with 0.5 μM TW-37 one hour prior to treatment with 0.4 μM vemurafenib for 72 h. Cell survival was evaluated spectrophotometrically. The experiment was performed at least three times independently, each time with four replicates and the data is expressed as mean ± S.D. *p < 0.05 when compared with control. #, *p* < 0.05 when compared with vemurafenib treatment. In a similar experiment, lysates of **I.** A375 and **J.** SK-MEL-28 cells were subjected to western blotting and analyzed for Mcl-1, cleaved caspase 3 and cleaved PARP. Each experiment was performed at least three independent times. β actin was used as loading control in all the western blot experiments.

To choose the working concentration, we initially determined concentration-dependent effects of vemurafenib using cell viability assay. We used A375 and SK-MEL-28 melanoma cells, both of which harbor BRAF mutation at V600E. The IC_50_ of vemurafenib in A375 and SK-MEL-28 cells at 72 hours of treatment were 0.1 μM and 0.075 μM respectively (Fig. 1A–1B). Based on these results, A375 and SK-MEL-28 cells were treated with 0.1, 0.2 and 0.4 μM vemurafenib for 72 hours (Fig. 1C). Our results showed a significant upregulation of Mcl-1 expression upon vemurafenib treatment in both the cell lines (Fig. 1C). Vemurafenib treatment increased the expression of Mcl-1 by 4.7, 5.2 and 4.5 fold in A375 cells and by 10, 11 and 14 fold in SK-MEL-28 cells at 0.1, 0.2 and 0.4 μM respectively (Fig. 1C). However, there was no significant change in the expression of Bcl-2 and Bcl-XL upon vemurafenib treatment (Fig. 1C). We further treated eight other melanoma cell lines with 0.4 μM vemurafenib and observed significant upregulation of Mcl-1 in all the cell lines (Supp Fig. 1). The fold increase in Mcl-1 expression in all the cell lines induced by single treatment of vemurafenib is shown in Fig. 1D.

Although the viability of A375 and SK-MEL-28 cells treated with 0.4 μM vemurafenib (4XIC_50_) was reduced by 60%, a remarkable increase in Mcl-1 expression was observed (Fig. 1C). These observations were intriguing and indicated that the increase in Mcl-1 expression we observed was perhaps coming from the remaining 40% of live attached cells that were resistant to vemurafenib. We therefore separated attached and floating cells after vemurafenib treatment and compared the levels of Mcl-1 by western blotting. Our results showed that there was a diminished expression of Mcl-1 in the dead floating cells (Figs. 1E–1F). In contrast, cells that survived upon vemurafenib treatment had significant upregulation of Mcl-1 as compared to control cells indicating that expression of Mcl-1 perhaps protected the cells from the cytotoxic effects of vemurafenib (Figs. 1E–1F).

### Mcl-1 inhibitor enhances the growth suppressive effects of vemurafenib

Since we observed that the cells that survived after vemurafenib treatment had significant upregulation of Mcl-1, we wanted to see whether TW-37, a Mcl-1 inhibitor, enhances vemurafenib mediated growth suppression. Vemurafenib (0.4 μM) treatment reduced the viability of A375 and SK-MEL-28 cells by 48% and 55% respectively (Figs. 1G–1H). TW-37 alone decreased the viability of A375 and SK-MEL-28 cells by 40% and 58% respectively (Figs. 1G–1H). However, combination of vemurafenib and TW-37 treatment reduced the cell survival by 85% and 79%, which was significantly higher than any of the single treatments (Figs. 1G–1H). These observations correlated with our western blot results. Vemurafenib failed to stimulate Mcl-1 when co-treated with TW-37 (Figs. 1I–1J). The combination treatment significantly induced the cleavage of caspase 3 and PARP, which was higher than any of the individual treatments, indicating apoptosis (Figs. 1I–1J).

### Vemurafenib resistant melanoma cells exhibit Mcl-1 overexpression

**Figure 2:**
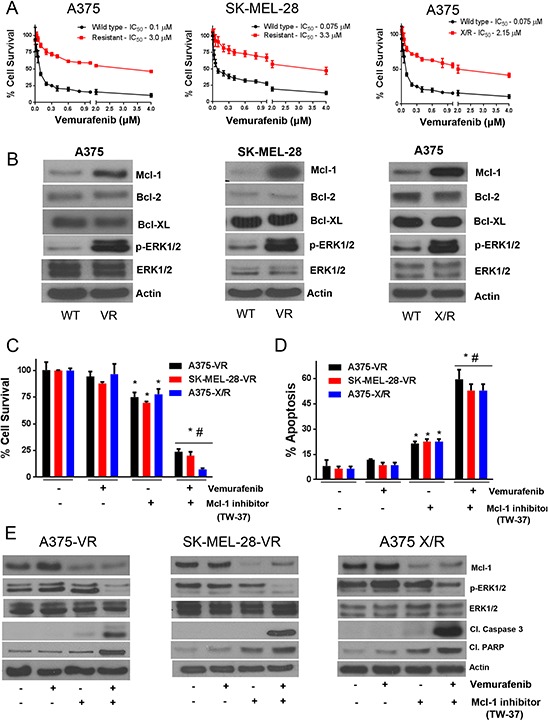
Vemurafenib resistant melanoma cells exhibit Mcl-1 overexpression **A.** A375, A-375-VR, SK-MEL-28, SK-MEL-28-VR and A375X/R cells were treated with various concentrations of vemurafenib for 72 hours following which the cell survival was analyzed by sulforhodamine B assay. The experiment was performed at least three times independently, each time with eight replicates and the data is expressed as mean ± S.D. **B.** Lysates of A375, A375-VR, SK-MEL-28, SK-MEL-28-VR and A375X/R were subjected to western blotting and analyzed for Mcl-1. Each experiment was performed three times independently. Mcl-1 inhibitor overcomes vemurafenib resistance in melanoma cells. **C–D.** A375-VR, SK-MEL-28-VR and A375X/R cells were treated with 0.5 μM TW-37 one hour prior to the treatment with 0.4 μM vemurafenib for 72 h after which cell survival or apoptosis was evaluated. The experiment was performed at least three times independently, each time with four replicates and the data expressed as mean ± S.D. *p < 0.05 when compared with control. #, *p* < 0.05 when compared with vemurafenib treatment. **E.** In a similar experiment, lysates of A375-VR, SK-MEL-28-VR and A375X/R cells were subjected to western blotting and analyzed for Mcl-1, p-ERK1/2, ERK1/2, cleaved caspase 3 and cleaved PARP. Each experiment was performed at least three independent times. β actin was used as a loading control in all the western blot experiments.

We further wanted to investigate the levels of Mcl-1 in the cells with vemurafenib resistance. Hence, we generated A375-VR and SK-MEL-28-VR vemurafenib resistant cell lines. The IC_50_ of vemurafenib in A375-VR and A375 X/R was 3.0 μM and 2.2 μM respectively, and that in SK-MEL-28-VR was 3.3 μM as compared to the IC_50_ of 0.1 μM and 0.075 μM in A375 and SK-MEL-28 parent (sensitive) cell lines (Fig. 2A). In all, we achieved 30–40 fold resistance to vemurafenib in these cell lines. The viability of resistant cells was not suppressed at the concentrations that suppressed more than 60% growth of the sensitive cell lines (Fig. 1G–1H and 2C). As expected, western blot results showed a massive increase in Mcl-1 expression in vemurafenib resistant cell lines (Fig. 2B). The fold increase of Mcl-1 expression in A375-VR and A375-X/R was 6.2 and 4.8 respectively, and that in SK-MEL-28-VR was 10.1, as compared to respective sensitive cells (Fig. 2B). Moreover, there was also a significant increase in the phosphorylation of ERK1/2 in all the resistant cell lines (Fig. 2B). We did not observe any significant difference in the expression of Bcl-2 and Bcl-XL between the wild type and resistant cell lines (Fig. 2B).

### Mcl-1 inhibitor overcomes vemurafenib resistance in melanoma cells

Vemurafenib, at a concentration of 0.4 μM, exhibited negligible effect on the survival of A375-VR cells (Fig. 2C). The survival of A375-VR cells was decreased by 25% when treated with TW-37 (Fig. 2C). However, when these resistant cells were treated with both TW-37 and vemurafenib, the survival of A375-VR cells was decreased significantly by 80% (Fig. 2C). Similarly, in SK-MEL-28-VR cells, vemurafenib showed minimal effect but when combined with TW-37, the survival of resistant cells was suppressed by about 80% (Fig. 2C). TW-37 treatment alone reduced the growth of SK-MEL-28-VR cells by 25% only (Fig. 2C). The enhanced efficacy of vemurafenib when combined with TW-37 can be attributed to the inhibition of Mcl-1, rendering the cells sensitive to vemurafenib. Similar results were observed in A375-X/R cells (Fig. 2C). These observations were further validated by apoptosis assay. Vemurafenib treatment failed to induce apoptosis in all the vemurafenib resistant cell lines (Fig. 2D). There was a 2-fold increase in apoptosis when these cells were treated with TW-37 (Fig. 2D). Finally, when these cells were treated with a combination of TW-37 and vemurafenib, there was more than 5-fold increase in apoptosis (Fig. 2D). Our western blot results showed marked expression of Mcl-1 in vemurafenib resistant untreated cells, whereas vemurafenib treatment further increased the expression of Mcl-1 in all the resistant cell lines and no cleavage of caspase 3 was observed in all the cell lines (Fig. 2E). However, vemurafenib in combination with TW-37, which significantly inhibited Mcl-1, increased the cleavage of caspase 3 and PARP, indicating apoptosis (Fig. 2E). Activation of ERK1/2 was also observed in vemurafenib resistant cell lines (Fig. 2E). Furthermore, vemurafenib treatment did not have any effect on phosphorylation of ERK1/2 at Thr202/Tyr204 but when combined with TW-37, ERK1/2 phosphorylation was suppressed (Fig. 2E). No change in the protein level of ERK1/2 was observed by individual or combination treatment (Fig. 2D). These results clearly indicated the role of Mcl-1 in inducing resistance to vemurafenib in malignant melanoma cells.

### Mcl-1 overexpression reduce the sensitivity of melanoma cells to vemurafenib

**Figure 3:**
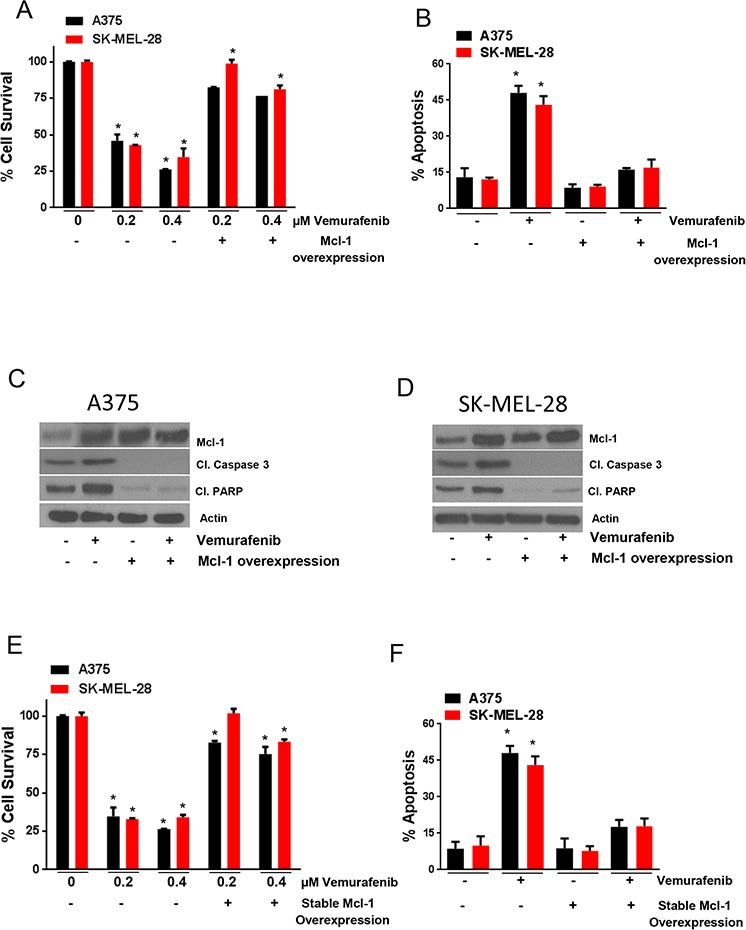
Mcl-1 overexpression reduces the sensitivity of melanoma cells to vemurafenib **A–B.** A375 and SK-MEL-28 untransfected or transfected with Mcl-1 plasmid were treated with 0.2 μM and 0.4 μM vemurafenib for 72 hours. The cell survival was evaluated by sulforhodamine B assay and apoptosis was evaluated by Annexin V/FITC assay. The experiment was performed at least three times independently, each time with four replicates and the data is expressed as mean ± S.D. *p < 0.05 when compared with control. In a similar experiment, **C.** A375 and **D.** SK-MEL-28 cells untransfected or transfected with Mcl-1 plasmid were treated with 0.4 μM vemurafenib for 72 hours. Following the treatment, the lysates were subjected to western blotting and analyzed for Mcl-1, cleaved caspase 3 and cleaved PARP. β actin was used as a loading control. Each experiment was performed at least three times independently. **E.** A375 and A375-Mcl-1+/+ or SK-MEL-28 and SK-MEL-28-Mcl-1+/+ were treated with 0.2 μM and 0.4 μM vemurafenib for 72 hours and cell survival was evaluated. **F.** A375 and A375-Mcl-1+/+ or SK-MEL-28 and SK-MEL-28-Mcl-1+/+ were treated with 0.4 μM vemurafenib for 72 hours and apoptosis was evaluated. The experiment was performed at least three times independently, each time with four replicates and the data is expressed as mean ± S.D. *p < 0.05 when compared with control.

To further characterize the role of Mcl-1 in vemurafenib resistance, we overexpressed Mcl-1 in A375 and SK-MEL-28 cells by transfecting the cells with Mcl-1 overexpressing plasmid. The survival of A375 cells was reduced by about 55% and 75% and SK-MEL-28 cells by 57% and 67%, when treated with 0.2 μM and 0.4 μM vemurafenib respectively (Fig. 3A). However, upon Mcl-1 overexpression, the effect of vemurafenib was significantly reduced in both the cell lines (Fig. 3A). For example, the survival of Mcl-1 overexpressing A375 was reduced merely by 18% and 24% when treated with 0.2 μM and 0.4 μM of vemurafenib respectively (Fig. 3A). Similarly, SK-MEL-28 cells overexpressing Mcl-1 were completely resistant when treated with 0.2 μM vemurafenib whereas 0.4 μM reduced the survival of cells by only 18% (Fig. 3A). We also tested the effect of Mcl-1 overexpression on apoptosis induced by vemurafenib. There was about 4-fold induction of apoptosis when A375 and SK-MEL-28 cells were treated with vemurafenib (Fig. 3B). Upon Mcl-1 overexpression, the apoptosis induced by vemurafenib was completely abrogated (Fig. 3B). These results were further supported by our western blot results. Upon vemurafenib treatment, there was a marked cleavage of caspase 3 and PARP in both the cell lines, which was completely diminished upon Mcl-1 overexpression (Figs. 3C–3D).

To further establish a connection between Mcl-1 and vemurafenib resistance, we generated A375-Mcl-1+/+ and SK-MEL-28-Mcl-1+/+ cell lines exhibiting stable overexpression of Mcl-1, and then evaluated the effect of vemurafenib in these cell lines. As expected, these cells behaved very similar to the resistant cells when treated with vemurafenib. The survival of A375-Mcl-1+/+ cells was reduced by only 20% and 25% when treated with 0.2 μM and 0.4 μM vemurafenib respectively (Fig. 3E). In SK-MEL-28-Mcl-1+/+ cells, no change was observed when treated with 0.2 μM vemurafenib but about 15% reduction in cell survival was observed when treated with 0.4 μM vemurafenib (Fig. 3E). In contrast, both the respective wild type (parent) cell lines were highly sensitive to vemurafenib treatment (Fig. 3E). Vemurafenib treatment was able to induce a modest 2 fold increase in apoptosis in both the melanoma cell lines with stable Mcl-1 overexpression in contrast to 6 fold induction of apoptosis in the respective parent cell lines (Fig. 3F). These results established the involvement of Mcl-1 in the induction of acquired resistance to vemurafenib in melanoma cells.

### Silencing Mcl-1 reverses vemurafenib resistance

**Figure 4:**
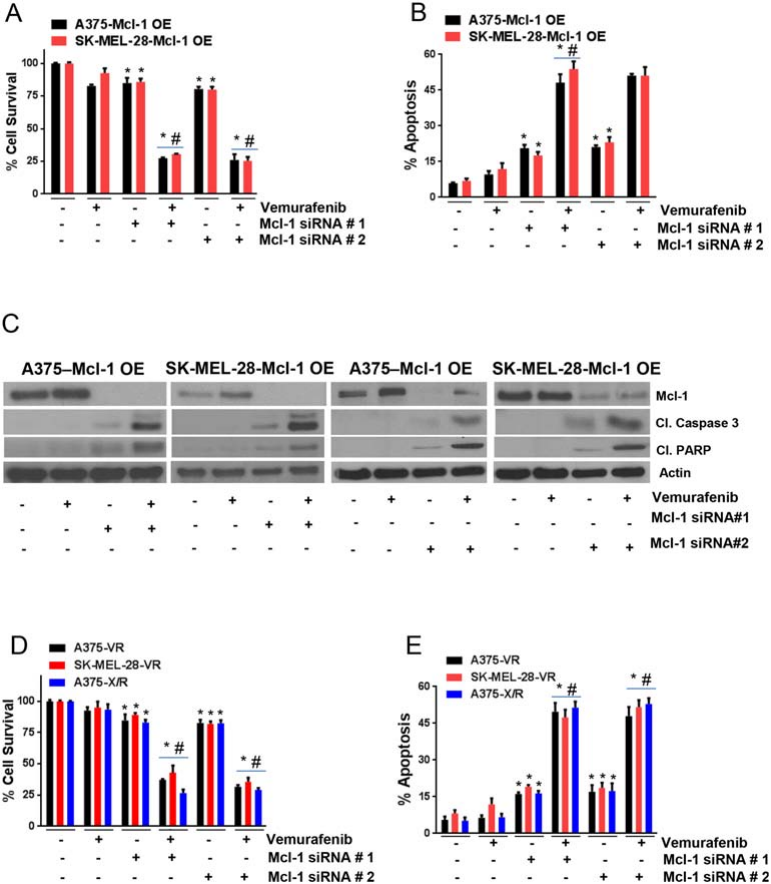

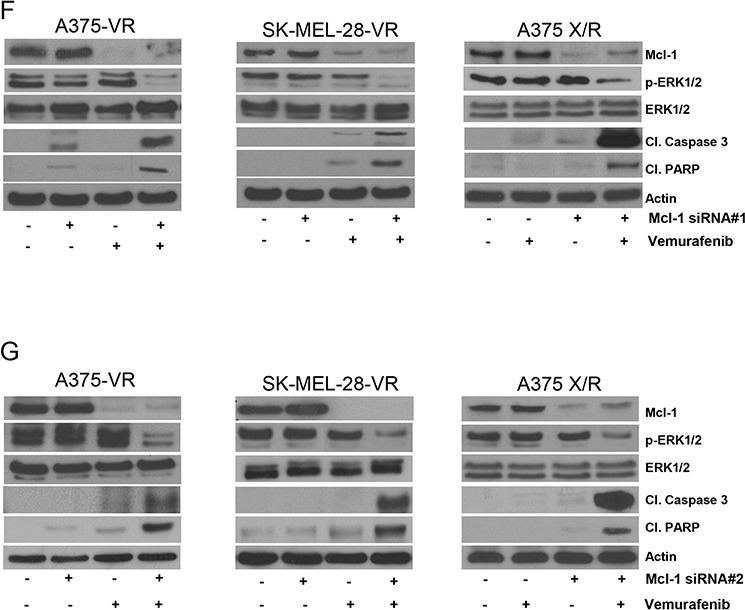
Silencing Mcl-1 reverses vemurafenib resistance Mcl-1 was silenced by siRNA#1 or siRNA#2 in **A–B.** A375-Mcl-1+/+, SK-MEL-28-Mcl-1+/+, **D–E.** A375-VR, SK-MEL-28-VR and A375-X/R after which the cells were treated with 0.4 μM vemurafenib for 72 hours. The cell survival was evaluated by sulforhodamine B assay and apoptosis was evaluated by Annexin V/FITC assay. The experiment was performed at least three times independently, each time with four replicates and the data is expressed as mean ± S.D. *p < 0.05 when compared with control. #, *p* < 0.05 when compared with vemurafenib treatment. In a similar experiment, after silencing Mcl-1 by siRNA#1 or Mcl-1 siRNA#2, **C.** A375-Mcl-1+/+, SK-MEL-28-Mcl-1+/+ and **F–G.** A375-VR, SK-MEL-28-VR and A375-X/R cell lysates were prepared and the protein was subject to western blotting and analyzed for Mcl-1, p-ERK1/2, ERK1/2, cleaved caspase 3 and cleaved PARP. Each experiment was performed at least three times independently. β actin was used as a loading control.

To examine whether silencing Mcl-1 could overcome vemurafenib resistance in melanoma cells, Mcl-1 was silenced using two different siRNAs, which were denoted as siRNA#1 and siRNA#2. The sequences of siRNAs are given in Supplemental Table 1. We first tested the effect of Mcl-1 silencing in A375-Mcl-1+/+ and SK-MEL-28-Mcl-1+/+ cells, since the response of these cells to vemurafenib was analogous to the resistant cell lines (A375-VR and SK-MEL-28-VR). Almost 100% silencing of Mcl-1 was achieved with both the siRNAs in both the Mcl-1 overexpressing cell lines (Fig. 4C). As mentioned above, vemurafenib treatment had very minimal effect on A375-Mcl-1+/+ cells whereas SK-MEL-28-Mcl-1+/+ cells were completely resistant (Fig. 4A). However, upon silencing of Mcl-1by siRNA#1 or siRNA#2, vemurafenib suppressed the survival of A375-Mcl-1+/+ cells by 73% and that of SK-MEL-28-Mcl-1+/+ cells by 70% (Fig. 4A). Likewise, combination of vemurafenib with Mcl-1 siRNA#1 and Mcl-1 siRNA#2 induced 5 fold apoptosis in both the Mcl-1 overexpressing cell lines (Fig. 4B). These observations were further supported by our western blot results. With vemurafenib treatment, there was no change in the cleavage of caspase 3 and PARP as compared to control in both the Mcl-1 +/+ cell lines (Fig. 4C). However, when Mcl-1 was silenced and the cells were treated with vemurafenib, significant apoptosis was induced as depicted by cleavage of caspase 3 and PARP (Fig. 4C).

Next, we sought to determine the effect of vemurafenib in A375-VR, A375-X/R and SK-MEL-28-VR cells after silencing Mcl-1, as these cells expressed very high levels of Mcl-1. About 85–95% Mcl-1 silencing was achieved by both the siRNAs in all the resistant cell lines (Fig. 4F–4G). Upon Mcl-1 silencing, there was a significant reduction in cell survival in all the resistant cell lines when treated with vemurafenib. The survival of A375-VR, A375-X/R and SK-MEL-28-VR cells when treated with vemurafenib was decreased by 65%, 75% and 55% respectively when Mcl-1 was silenced in these resistant cells (Fig. 4D). Moreover, the sensitivity of Mcl-1 silenced resistant cells to vemurafenib was very similar to that of parent (sensitive) cells that we observed earlier (Fig. 1A–1B). Additionally, the combination of vemurafenib and Mcl-1 siRNA#1 or Mcl-1 siRNA#2 induced 5-fold apoptosis in all the resistant cell lines, which were initially unresponsive to vemurafenib treatment (Fig. 4E). These results were further supported by western blotting data (Fig. 4F–4G). Vemurafenib treatment induced very minimal cleavage of caspase 3 and PARP in resistant cell lines (Fig. 4F–4G). Nonetheless, treatment of cells with vemurafenib after silencing of Mcl-1 induced notable cleavage of caspase 3 and PARP (Fig. 4F–4G). Furthermore, vemurafenib treatment did not have any effect on p-ERK1/2 but when combined with Mcl-1 siRNA, phosphorylation of ERK1/2 was significantly suppressed (Fig. 4F–4G). The exact mechanism by which Mcl-1 inhibition regulates phosphorylation of ERK1/2 in our model is not clear and requires further investigation. The expression of ERK1/2 remained unchanged by all the treatments (Figs. 4F–4G). These results established that inhibition of Mcl-1 completely overcomes the acquired resistance to vemurafenib in melanoma cells.

### Inhibiting Mcl-1 suppresses the growth of melanoma tumors resistant to vemurafenib

**Figure 5:**
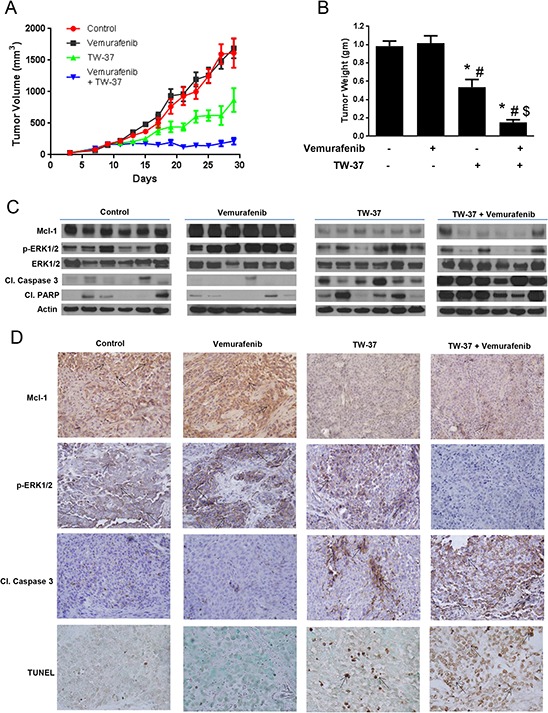
Inhibiting Mcl-1 suppresses the growth of melanoma tumors resistant to vemurafenib **A.** A375-VR cells were injected subcutaneously in female athymic nude mice. Once the tumor volume reached 150 mm^3^, mice were randomly divided into 4 groups (*n* = 7 in each group) and the treatment was started as described under ‘Material and Method’ section. Group 1 mice treated with vehicle served as control, group 2 mice were treated with vemurafenib, group3 mice were treated with TW-37 and group 4 mice were treated with a combination of vemurafenib and TW-37. Tumor volumes were measure thrice a week by vernier calipers and the values were plotted as Mean ± S.E.M. **B.** At day 30, mice were sacrificed, tumors were extracted and weighed. The values are plotted as mean ± S.D. *p < 0.05 as compared to control. #, *p* < 0.05 as compared to vemurafenib treated group. $, *p* < 0.05 as compared to TW-37 treated group. **C.** Tumor lysates from 6 mice were subjected to western blotting and analyzed for Mcl-1, p-ERK1/2, ERK, cleaved caspase 3 and cleaved PARP. β actin was used as loading control. **D.** Formalin fixed paraffin embedded tumor sections were subjected to immunohistochemistry and TUNEL assay. Representative images of the tumor sections stained with TUNEL, Mcl-1, p-ERK1/2 and cleaved caspase 3.

**Figure 6:**
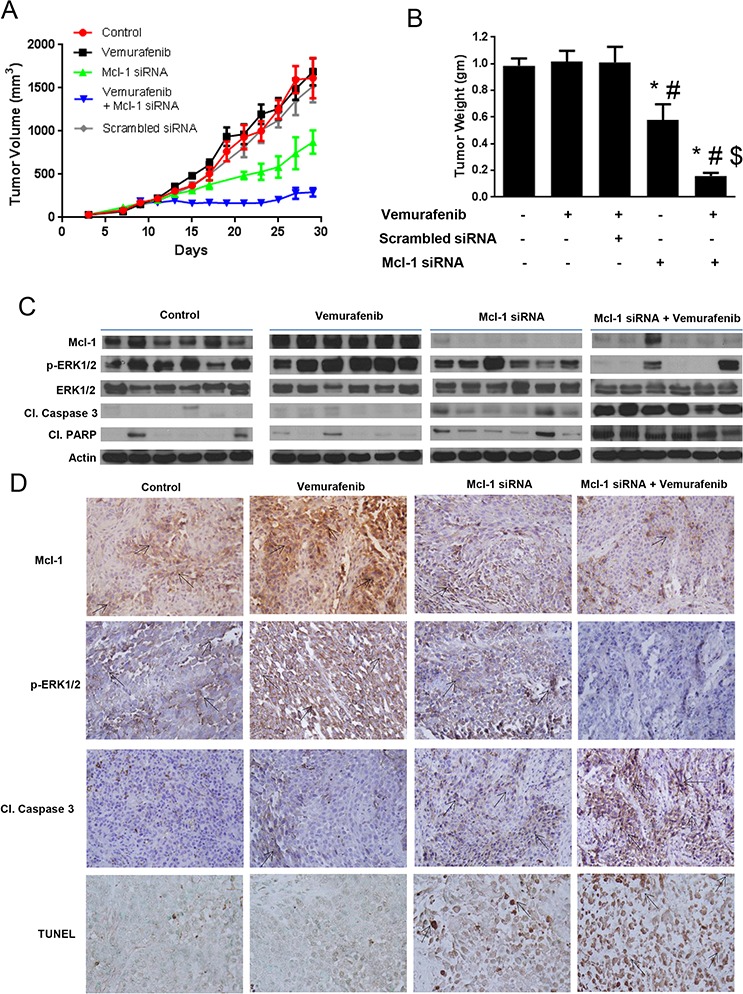
Silencing Mcl-1 suppresses the growth of melanoma tumors resistant to vemurafenib **A.** A375-VR cells were injected subcutaneously in female athymic nude mice. Once the tumor volume reach 150 mm^3^, the mice were randomly divided into 5 groups (*n* = 6 in each group) and the treatment was started as described under ‘Material and Method’ section. Tumor volumes were measure thrice a week by vernier calipers and the values were plotted as Mean ± S.E.M. **B.** At day 30, mice were sacrificed, tumors were extracted and weighed. The values are plotted as mean ± S.D. *p < 0.05 as compared to control. #, *p* < 0.05 as compared to vemurafenib treated group. $, *p* < 0.05 as compared to TW-37 treated group. **C.** Tumor lysates from 6 mice were subjected to western blotting and analyzed for Mcl-1, p-ERK1/2, ERK1/2, cleaved caspase 3 and cleaved PARP. β actin was used as a loading control. **D.** Formalin fixed paraffin embedded tumor sections were subjected to immunohistochemistry and TUNEL assay. Representative images of the tumor sections stained with TUNEL, Mcl-1, p-ERK1/2 and cleaved caspase 3.

Although it was very evident from our *in vitro* results that inhibition of Mcl-1 completely reversed the acquired resistance to vemurafenib in melanoma cells, it was of utmost importance to translate these observations *in vivo.* At day 30, the average tumor volume of the control group was 1613.5 ± 231.9 mm^3^ while that of vemurafenib treated group was 1688 ± 156.19 mm^3^ (Fig. 5A and 6A) indicating that the tumors did not respond to vemurafenib treatment at all. The tumor volume of the mice that were treated with TW-37 alone was 870 ± 187.8 mm^3^ demonstrating a 48% and 46% reduction in tumor growths as compared to the tumor volumes of the mice from control as well as vemurafenib treated group, respectively (Fig. 5A). Most importantly, mice that were treated with a combination of vemurafenib and TW-37 had significantly lower tumor volumes as compared to all the three groups (Fig. 5A). The average tumor volumes of these mice at the end of the experiment were 215.3 ± 51.6 mm^3^, showing a marked reduction in tumor growth by more than 85% as compared to control or vemurafenib treated group (Fig. 5A). In fact, the tumors did not grow much once the treatment started.

The average tumor volume of the mice that were treated with Mcl-1 siRNA alone at the end of the experiment was 875 ± 134.3 mm^3^, which was significantly lower than that of control and vemurafenib treated mice (Fig. 6A). The average tumor volume of the mice treated with scrambled siRNA was 1553 ± 650 mm^3^, showing no significant difference between the average volumes of the tumor of the mice treated with scrambled siRNA, vemurafenib or control mice (Fig. 6A). However, the average volume of the tumors in the mice that were treated with vemurafenib and Mcl-1 siRNA was 292 ± 48.12 mm^3^, showing a notable suppression of tumor growth by more than 80%, as compared to control or vemurafenib treated group (Fig. 6A).

At day 30, mice from all the groups were sacrificed and the tumors were removed and weighed. As shown in Fig. 5B, there was no difference in the tumor weight of control and vemurafenib treated mice. The weight of the tumor in TW-37 treated group was reduced by 47% as compared to control and 48% as compared to vemurafenib treated group (Fig. 5B). Notably, the tumor weight in the mice treated with TW-37 and vemurafenib was reduced by more than 85%, consistent with tumor volume data. The weight of the tumor in Mcl-1 siRNA treated group was reduced by 40% as compared to control and 43% as compared to vemurafenib treated group (Fig. 6B). Moreover, the tumor weight in the mice treated with Mcl-1 siRNA and vemurafenib was reduced by more than 85%, consistent with tumor volume data (Fig. 6B). These results clearly indicated that Mcl-1 overexpression led to vemurafenib resistance and that inhibition of Mcl-1 sensitized the vemurafenib resistant tumors to vemurafenib.

### Vemurafenib resistant tumors exhibit overexpression of Mcl-1

Upon termination of *in vivo* experiments, tumors were examined by western blotting and immunohistochemistry. The control tumors showed marked expression of Mcl-1 (Fig. 5 and 6C–6D). Interestingly, tumors from vemurafenib treated group had even higher expression of Mcl-1 than the tumors from control group (Fig. 5 and 6C–6D). The tumors from the mice that were treated with either Mcl-1 inhibitor (Fig. 5C–5D) or Mcl-1 siRNA alone (Fig. 6C–6D) had diminished expression of Mcl-1. Nonetheless, the tumors from the mice that were treated with a combination of vemurafenib with either Mcl-1 inhibitor or Mcl-1 siRNA had significantly lower expression of Mcl-1 as compared to the tumors from control or vemurafenib treated group (Fig. 5C–5D). We also examined the expression of cleaved caspase 3, cleaved PARP and p-ERK1/2 in these tumors. Tumors from control and vemurafenib group showed minimal cleavage of caspase 3 or PARP (Fig. 5 and 6C–6D). The tumors treated with Mcl-1 inhibitor or Mcl-1 siRNA showed modest cleavage of caspase 3 and PARP (Fig. 5 and 6C–6D). However, the tumors that were treated with the combination of vemurafenib with Mcl-1 inhibitor or Mcl-1 siRNA showed massive cleavage of caspase3 and PARP. Expression of p-ERK1/2 was evaluated to check the inhibition of MAPK pathway. In control and vemurafenib treated group, where there was high expression of Mcl-1, we also observed notable phosphorylation of ERK1/2 (Fig. 5 and 6C–6D). Treatment with Mcl-1 inhibitor or Mcl-1 siRNA had minimal effect on p-ERK1/2 expression (Fig. 5 and 6C–6D). However, upon combining vemurafenib with either Mcl-1 inhibitor or Mcl-1 siRNA, there was a substantial decrease in the phosphorylation of ERK1/2, hence, indicating the inhibition of MAPK pathway (Fig. 5 and 6C–6D). Nevertheless, the expression of ERK1/2 was not changed by any of the treatments (Fig. 5C and 6C). Finally, TUNEL staining exhibited significant staining in the tumors of the mice that were treated with a combination of vemurafenib and Mcl-1 inhibitor/siRNA, indicating apoptosis (Fig. 5D and 6D). Tumors from the mice treated with vemurafenib showed negligible or no TUNEL staining, which was similar to the tumors from control mice (Fig. 5D and 6D).

### Dabrafenib induces Mcl-1 expression in melanoma cells

**Figure 7:**
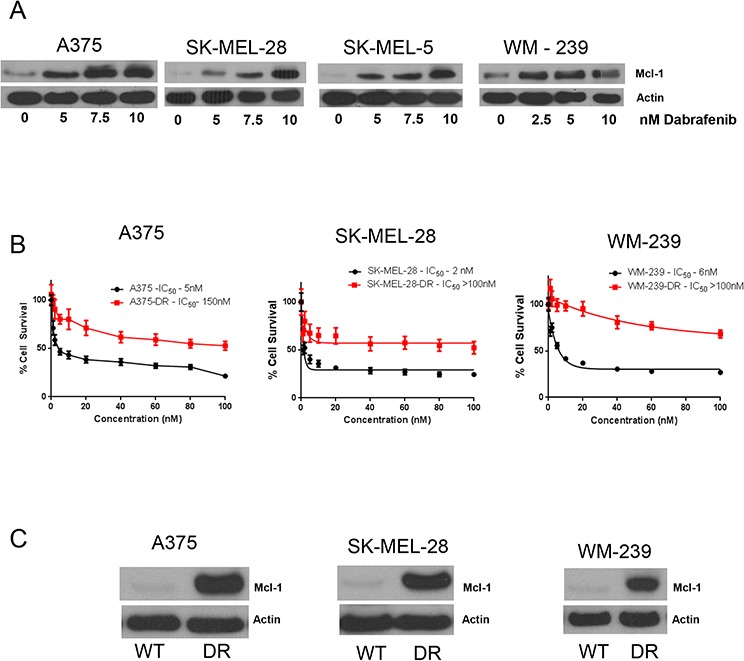
Dabrafenib induces Mcl-1 expression in melanoma cells **A.** A375, SK-MEL-28, SK-MEL-5 and WM-239 were treated with 5–10 nM dabrafenib for 72 hours. Following the treatment, Mcl-1 expression was analyzed by western blotting. **B.** Cytotoxicity of dabrafenib at 72 hours in wildtype and dabrafenib resistant A375-DR, SK-MEL-28-DR and WM-239-DR was evaluated by SRB assay. **C.** The expression of Mcl-1 in A375, SK-MEL-28 and WM-239 wild type cells and dabrafenib resistant cells was evaluated by western blotting. β actin was used as a loading control in all the western blot experiments. Each experiment was performed at least three times independently.

While we were working with vemurafenib, dabrafenib, another BRAF inhibitor was approved for the treatment of BRAF mutant melanoma. Hence, we wanted to see whether the role of Mcl-1 in drug resistance was specific to vemurafenib or applicable to other BRAF inhibitors as well. We treated A375, SK-MEL-28, WM239 and SK-MEL-5 cells with 2.5, 5 and 10 nM dabrafenib for 72 hours. Similar to vemurafenib, we observed that dabrafenib treatment also induced massive Mcl-1 expression at all the three concentrations (Fig. 7A). We then generated dabrafenib resistant A375, SK-MEL-28 and WM-239 cells, which were denoted as A375-DR, SK-MEL-28-DR and WM-239-DR. The IC_50_ of dabrafenib in A375, SK-MEL-28 and WM-239 was 5nM, 2nM and 6nM respectively, and that of A375-DR, SK-MEL-28-DR and WM-239-DR was greater than 100 nM indicating more than 30-fold resistance (Fig. 7B). As expected, dabrafenib resistant melanoma cells showed marked upregulation of Mcl-1 as compared to the respective wild type (sensitive) cells (Fig. 7C). The fold-increase in Mcl-1 expression in A375-DR, SK-MEL-28-DR and WM-239-DR was 14.2, 19.4 and 12.9 respectively (Fig. 7C).

### Dabrafenib-Trametinib combination induces Mcl-1 expression in melanoma cells

**Figure 8:**
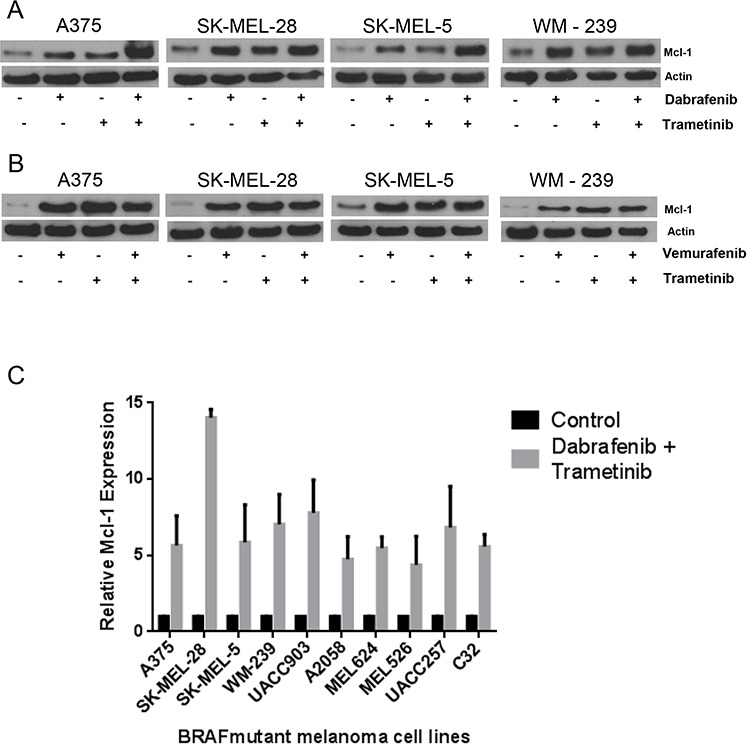
Dabrafenib-Trametinib combination induces Mcl-1 expression in melanoma cells causing drug resistance A375, SK-MEL-28, SK-MEL-5 and WM-239 were treated **A.** 10 nM dabrafenib and 2nM trametinib and **B.** 0.4 μM vemurafenib and trametinib for 72 hours. Following the treatment, Mcl-1 expression was analyzed by western blotting. β actin was used as a loading control in all the western blot experiments. **C.** Ten mutant BRAF melanoma cell lines were treated with a combination of dabrafenib (10nM) and trametinib (2 nM) for 72 hours. The protein was collected, subjected to western blotting and analyzed for Mcl-1 expression. Fold increase in Mcl-1 expression was calculated by densitometric analysis. Actin was used as a loading control. Each experiment was performed at least three times independently.

Early 2014, FDA approved a combination of BRAF inhibitor (dabrafenib) and MEK1/2 inhibitor (trametinib) for the treatment of late stage malignant melanoma, due to ineffectiveness of vemurafenib monotherapy. Hence, we wanted to know whether the role of Mcl-1 in drug resistance was specific to BRAF inhibitors or applicable to the combination of BRAF and MEK1/2 inhibitors as well. We treated the cells with BRAF inhibitors (dabrafenib or vemurafenib) and MEK 1/2 inhibitor (trametinib) alone as well as in combination. As observed earlier, dabrafenib at a concentration of 10 nM significantly induced the expression of Mcl-1 in A375, SK-MEL-28, SK-MEL-5 and WM-239 cells (Fig. 8A). Notably, trametinib treatment also induced Mcl-1 expression in all the melanoma cell lines tested (Fig. 8A–8B). Finally, treatment of melanoma cells with a combination of vemurafenib or dabrafenib with trametinib also caused remarkable induction of Mcl-1 which was more as compared to individual treatments (Fig. 8A–8B). Induction of Mcl-1 by treatment with a combination of dabrafenib and trametinib was investigated in a panel of ten BRAF mutant cell lines. Treatment of these cells with the combination of dabrafenib and trametinib induced significant expression of Mcl-1 in all the cell lines (Supp. Fig. 2). The fold increase in Mcl-1 expression in response to combination treatment in each cell line is shown in Fig. 8C. These results indicated that BRAF inhibitors alone as well as in combination with MEK1/2 inhibitors induce Mcl-1 expression.

### Mcl-1 overexpressing melanoma cells are resistant to combined BRAF inhibitor and MEK1/2 inhibitor treatment

**Figure 9:**
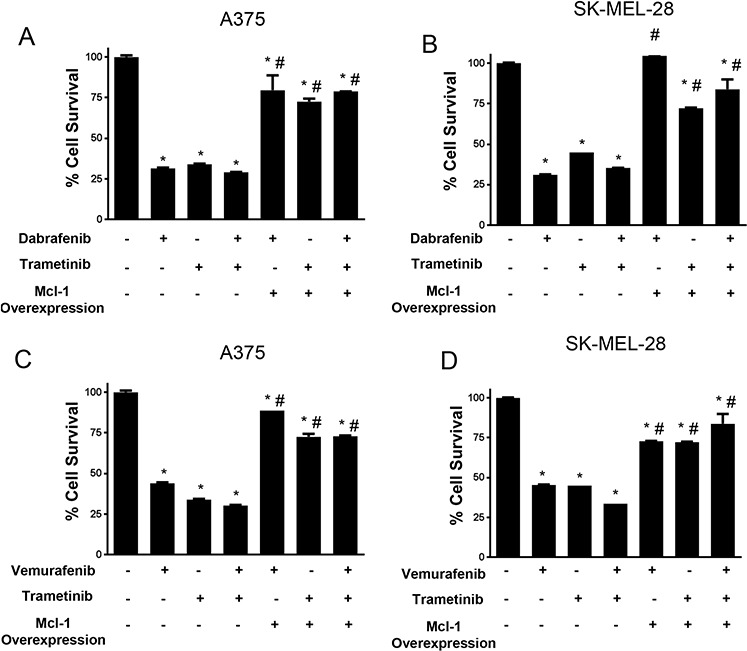
Mcl-1 overexpression reduces the sensitivity of melanoma cells to BRAF inhibitor and MEK inhibitor combination treatment **A.** A375 and **B.** SK-MEL-28 untransfected or transfected with Mcl-1 plasmid were treated with 10 nM dabrafenib or 2 nM trametinib for 72 hours. **C.** A375 and **D.** SK-MEL 28 cells untransfected or transfected with Mcl-1 plasmid were treated with 0.4 μM vemurafenib or 2 nM trametinib for 72 hours. The cell survival was evaluated by sulforhodamine B assay. The experiment was performed at least three times independently, each time with four replicates and the data is expressed as mean ± S.D. *p < 0.05 when compared with control. #, *p* < 0.05 when compared to respective cells transfected with empty vector. Each experiment was performed at least three times independently.

Based on our results in Fig. 7 and 8A, we hypothesized that induction of Mcl-1 induces resistance to the combination treatment of dabrafenib and trametinib in melanoma cells. In order to test our hypothesis and to characterize the role of Mcl-1 in resistance to the combination treatment of BRAF inhibitor with MEK1/2 inhibitor, we evaluated the effect of Mcl-1 overexpression on the efficacy of these therapeutic regimens. Our results showed that Mcl-1 overexpression not only reduced the efficacy of either BRAF inhibitor or MEK1/2 inhibitor alone but also their combination (Fig. 9A–9D). For example, dabrafenib, trametinib or the combination reduced the survival of A375 and SK-MEL-28 cells by 60–70% (Fig. 9A–9B). However, upon Mcl-1 overexpression, the reduction in cell survival with any of the treatments was at the most 25% (Fig. 9A–9B). In SK-MEL-28, Mcl-1 overexpression completely blocked the effect of dabrafenib (Fig. 9B). Similar results were observed when Mcl-1 overexpressing melanoma cells were treated with a combination of vemurafenib and trametinib (Fig. 9C–9D).

### Tumor biopsy samples exhibit enhanced Mcl-1 expression in patients treated with BRAF inhibitors alone or in combination with MEK1/2 inhibitors

**Figure 10:**
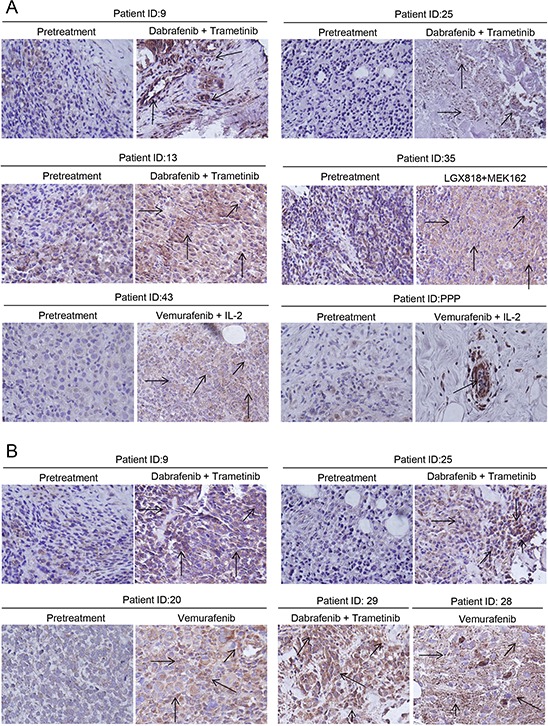
Tumor biopsy samples exhibited enhanced Mcl-1 expression in patients treated with BRAF inhibitor alone or in combination with MEK1/2 inhibitor Immunohistochemistry was performed to analyze Mcl-1 in tumor biopsy samples from patients **A.** before the treatment with BRAF inhibitor or combination and on BRAF inhibitor and **B.** before the treatment followed by progression biopsy. The patients were treated with either BRAF inhibitors (vemurafenib, dabrafenib, LGX818) alone or in combination with BRAF inhibitors (trametinib, MEK162). The representative positive cells are shown by the arrow.

Next, we wanted to confirm our hypothesis by examining clinical specimens. The exact treatment regimen along with the response of patient to treatment is given in Table 1. Mcl-1 expression was evaluated in the biopsied tumor samples by immunohistochemistry. Our results showed that tumors that were biopsied from the patients treated with BRAF inhibitors had significantly higher expression of Mcl-1 as compared to the tumors from the same patient before any treatment (Fig. 10A). Since the patients initially responded to the treatment, some of the biopsy samples had very few tumor cells. However, tumor cells that survived the treatment exhibited overexpression of Mcl-1 (Fig. 10A). Moreover, the tumor samples from patients (9, 13, 25, and 29) which received both dabrafenib and trametinib also demonstrated elevated expression of Mcl-1 (Fig. 10A). Furthermore, progression biopsy revealed enhanced expression of Mcl-1 as compared to the pretreated tumors from the same patient (Fig. 10B). Hence, these results showed that treatment with BRAF inhibitor alone or in combination with MEK1/2 inhibitor caused Mcl-1 overexpression, which correlated with relative resistance.

**Table 1:** Patient samples

Patient ID	RX	Response (RECIST)	Time to Progression (months)
**9**	dabrafenib + trametinib	PR (−45%)	7
**13**	dabrafenib + trametinib	PR (−57.9%)	9,stroke
**35**	LGX818 + MEK162	SD (−22.8%)	stopped after 7 months, PD at 10 months
**43**	BRAFi + IL2	CR (−81.5%)	13.4
**20**	vemurafenib	PR (−51.2%)	5
**28**	vemurafenib	SD	22
**29**	dabrafenib + trametinib	PR (−79%)	9
**PPP**	BRAFi + IL2	PR (−72.6%)	Ongoing
**25**	dabrafenib + trametinib	PR (−64%)	3

Patients with metastatic melanoma containing BRAF^V600E^mutation (confirmed by genotyping) were enrolled on clinical trials for treatment with a BRAF inhibitor (vemurafenib) or combined BRAF + MEK inhibitor (dabrafenib + trametinib). Listed are patient ID, treatment, maximum response (RECIST), time to progression (months).

## DISCUSSION

All the melanoma cell lines tested in the present study were highly sensitive to the treatment with BRAF inhibitors (vemurafenib and dabrafenib) alone or in combination with MEK1/2 inhibitor (trametinib). In spite of this, it was surprising to see a significant upregulation of Mcl-1 in the surviving cells following a single treatment with BRAF inhibitors. Our previous study did not observe any significant change in the expression of Mcl-1 with BRAF inhibitor treatment [26]. However, the expression of Mcl-1 in aforementioned study was tested only after 24 hours of treatment in contrast to 72 hours treatment in the current study. This suggests that Mcl-1 expression was induced upon longer duration of treatment. Moreover, the dead floating cells which responded to vemurafenib treatment had diminished Mcl-1 expression, consistent with previous reports indicating that inhibition of oncogenic BRAF led to inhibition of Mcl-1 [27–29]. Subsequently, in the current study, the cells resistant to BRAF inhibitors exhibited enhanced expression of Mcl-1. This provided initial evidence that overexpression of Mcl-1 might be responsible for acquired resistance to BRAF inhibitors in melanoma cells.

Induction of Mcl-1 with the treatment with BRAF inhibitors may indicate activation of other pathways post treatment leading to upregulation of Mcl-1. Expression of Mcl-1 is promoted by various transcription factors like STATs, cAMP response elements and NFκB [16]. Recent studies have correlated the activation of Src and STAT-3 with vemurafenib resistance [30, 31]. Both these pathways can directly or indirectly stimulate Mcl-1 expression. Moreover, activation of other unknown pathways may also cause induction of Mcl-1 expression. Nonetheless, targeting upstream molecules may provide little benefit to overcome vemurafenib resistance as compared to targeting Mcl-1 directly, as Mcl-1 is the ultimate downstream molecule and directly responsible for causing resistance to BRAF inhibitors. Additionally, targeting upstream molecules for therapy often fails as the intermediary molecules often get mutated or influenced by cross talk with other survival pathways resulting in the ineffectiveness of therapy [32–34].

Our studies provided several *in vitro* and *in vivo* evidences to prove this and established Mcl-1 as the major culprit in inducing resistance to BRAF inhibitors in melanoma. Wild type A375 and SK-MEL-28 melanoma cells completely lost sensitivity to BRAF inhibitors alone or in combination with MEK1/2 inhibitors upon Mcl-1 overexpression and transformed them into resistant cells.

The involvement of Mcl-1 in resistance to BRAF inhibitors was strengthened when vemurafenib resistant A375-VR or SK-MEL-28-VR cells were treated with the combination of vemurafenib with TW-37 (Mcl-1 inhibitor) or Mcl-1 siRNAs, and the combination treatment markedly induced apoptosis in both the cell lines which were earlier resistant to vemurafenib treatment. Moreover, the combination of vemurafenib with TW-37 or Mcl-1 siRNA dramatically inhibited the growth of A375-VR tumors, which exhibited complete resistance to vemurafenib treatment. Surprisingly, even though TW-37 or Mcl-1 siRNA did not have any effect on the phosphorylation of ERK1/2, their combination with vemurafenib led to decrease in the levels of p-ERK1/2. This may indicate that in presence of Mcl-1 inhibitor, vemurafenib was effectively controlling the MAPK pathway. However, the exact mechanism by which phosphorylation of ERK1/2 was suppressed require further investigation.

TW-37 is a BH-3 mimetic which binds to Mcl-1 and Bcl-2 with a Ki of 260 and 290 nM respectively and consequently induces caspase activity. TW-37 binds to Bcl-XL with a 5 fold lower affinity (Ki≈1200 nM) [35, 36]. Therefore, in order to inhibit Bcl-XL, cells should be treated with at least 5 fold higher concentration of TW-37 as compared to that used in the current study, sufficient to inhibit Mcl-1 but not Bcl-XL [37]. We observed that treatment of melanoma cells with BRAF inhibitors did not induce Bcl-2 expression. Moreover, there was no difference in the expression of Bcl-2 between the wild type and resistant melanoma cells. Besides, previous studies have shown reduced expression of Bcl-2 in melanoma as compared to melanocytes and benign nevi [38–42], ruling out the involvement of Bcl-2 in our study. Furthermore, the concentrations of TW-37 used for this study were not able to inhibit the expression of Bcl-XL *in vivo* (Supplemental Fig. 3). These factors somewhat eliminated the involvement of other antiapoptotic Bcl-2 family of proteins. More importantly, the *in vitro* and *in vivo* observations were confirmed by using Mcl-1 siRNA along with vemurafenib treatment, which further ruled out the involvement of other antiapoptotic Bcl-2 family proteins. In fact, two Mcl-1 siRNAs with different sequences were used to ensure target specificity and to eliminate off-target actions.

Finally, highly elevated expression of Mcl-1 in the biopsy samples obtained from the patients that were treated with or resistant to BRAF inhibitors like vemurafenib or dabrafenib alone or in combination with MEK1/2 inhibitor (trametinib) further confirmed that Mcl-1 may be responsible for induction of resistance to the entire class of BRAF inhibitors.

The translational consequences of these findings are significant. Our results conclusively establish that overexpression of Mcl-1 is critical for melanoma cell survival in the setting of BRAF inhibitor treatment and induce clinical resistance to BRAF inhibitors. To date, Mcl-1 targeted therapies have not been well established in the clinic, however based on this work and others, Mcl-1 is an obviously high valued target in oncology. Instead, the major so-called BH3-mimetics that are in clinical trials are those that inhibit Bcl-2 quite selectively (ABT-199) or more broadly (navitoclax, ABT-737), though even navitoclax only selectively inhibits Bcl-2, Bcl-x, Bcl-w and does not have any activity against Mcl-1. Due to the exciting preclinical data with Bcl2/Bcl-x inhibitors in combination with single-agent BRAF inhibitors [26, 43], combined BRAF targeted therapy with navitoclax as part of a CTEP study (P9466, NCT01989585) has commenced. Still, the prediction from our current and previous work is that patients with high Mcl-1 expression exists, either at baseline or by BRAF inhibitors, and these patients will be less likely to benefit from such a combination. Next important step is to develop clinical assay to measure Mcl-1 expression, so that patients likely to benefit from non-Mcl-1 targeting BH3 mimetics could be identified, ideally prior to therapy or early after the commencement of therapy. Beyond that, as the clinical development of Mcl-1 inhibitors proceed, an early look at combinations with BRAF targeted therapy will be critical.

Taken together, our results conclusively established that overexpression of Mcl-1 was responsible for the resistance to BRAF inhibitors; the process mediated by BRAF inhibitors itself. Mcl-1 targeted therapies will have significant impact on the patients with melanoma tumors refractory to BRAF inhibitors alone or in combination with MEK1/2 inhibitor. Although, currently there is no FDA approved Mcl-1 inhibitor, the process to discover clinically useful Mcl-1 inhibitors has well begun [24, 25, 44, 45]. Our laboratory is also aggressively working on developing novel Mcl-1 inhibitors as drugs.

## MATERIAL AND METHODS

### Chemicals

Vemurafenib, dabrafenib, trametinib and TW-37 were purchased from Selleck Chemicals (Houston, YX, USA). Mcl-1 antibody was purchased from Abcam (Cambridge, MA, USA). All the antibodies and Mcl-1 siRNAs were procured from Cell Signaling Technology Inc. (Danvers, MA, USA). Plasmid overexpressing Mcl-1 was acquired from Addgene (Cambridge, MA, USA). TUNEL assay kit was purchased from Calbiochem (San Diego, CA, USA)

### Cell culture

A375 was a kind gift from Dr. Tyler Wakenda (Rochester University, NY), which was originally purchased from ATCC (Manassas, VA, USA). SK-MEL-28 and WM-239 cells were purchased from ATCC. SK-MEL-5 was a kind gift from Dr. Randy Burd. The authenticity of these cell lines was confirmed by STR analysis at Texas Tech University Health Sciences Center core facility (Lubbock, TX, USA). All the cell lines were cultured in Eagle's Minimum Essential Medium (EMEM) supplemented with 10% fetal bovine serum. MEL624, MEL526, UACC903, UACC257, C32 and A2058 were grown in DMEM medium supplemented with 10% FBS and 1% penicillin/streptomycin and 2 mM L-glutamine, and maintained at 37°C in a humidified atmosphere at 5% CO_2_. These cell lines were obtained from cryopreserved collections at Massachusetts General Hospital, courtesy of H.Tsao.

### Generation of BRAF-inhibitor resistant cell lines

Vemurafenib resistant clones of A375 and SK-MEL-28 were generated by continuous exposure of cells to escalating concentrations of vemurafenib over a period of one year. The cells were treated with vemurafenib for 72 hours after which fresh media was added to the cells. The cells were allowed to recover for 24 hours after which they were again exposed to vemurafenib as shown in Supp. Tables 2–3. In all, cells were exposed weekly to two treatments of vemurafenib for 72 hours each with a 24 hour recovery period between the treatments. The initial concentration of vemurafenib used was 0.2 μM which was eventually increased to as high as 10 μM. Similarly, dabrafenib resistant cells were cultured by incubating the cells with increasing concentrations of the drug for a period of 3 months as described above. The fold resistance was intermittently evaluated by cell viability assay. Vemurafenib resistant cell lines were referred to as SK-MEL-28-VR or A375-VR and dabrafenib resistant cell lines were referred to as A375-DR, SK-MEL-28-DR or WM-239-DR. Another resistant cell line was generated from A375 xenograft-resistant (A375 X/R) tumors. Here, A375-VR cells were injected subcutaneously in nude mice. When palpable tumors were formed, the mice were orally treated with 30 mg/kg vemurafenib twice a day. After 30 days of treatment, cells from the tumors of mice treated with vemurafenib were isolated and cultured *in vitro*. These cells were named as A375 X/R cells.

### Cytotoxicity analysis

SK-MEL-28, A375, WM-239, SK-MEL-28-VR, A375-VR, A375-X/R, A375-DR, SK-MEL-28-DR and WM-239-DR cells were treated with various concentrations of vemurafenib or dabrafenib and cytotoxicity was performed by SRB assay as previously described by us [46, 47].

### Apoptosis assay

Annexin V/FITC assay was perform by flow cytometry after TW-37 treatments, Mcl-1 overexpression or Mcl-1 silencing according to manufacturer's protocol (BD Biosciences, San Diego, CA, USA) as previously described by us [48].

### TW-37 treatment

SK-MEL-28, A375, SK-MEL-28-VR, A375-VR and A375-X/R were plated in a six-well plate at a density of 0.3 × 10^6^ cells/well and left overnight to attach. Next day, cells were treated with 500nM TW-37 for one hour followed by treatment with 0.4 μM vemurafenib for 72 hours. Cells were collected and processed for SRB assay, apoptosis assay or western blotting.

### Mcl-1 overexpression

A375 or SK-MEL-28 cells were transiently or stably transfected with plasmid overexpressing Mcl-1 by nucleofection kit (Lonza, Basel, Switzerland) according to manufacturer's protocol and previously described by us [49]. Briefly, 2 × 10^6^ cells were suspended in a reaction mixture from the kit specific to the cell line (Kit V for A375 and Kit R for SK-MEL-28 cells). The cells were transferred to the cuvettes and electroporated using the nucleofector instrument (Amaxa, Cologne, Germany). To achieve stable overexpression, after transfection, cells were exposed to puromycin with an initial concentration of 1 μg/ml, which was gradually increased to 5 μg/ml. The resistant colonies were picked by colony picking cylinder and were cultured in presence of puromycin (5 μg/ml). Stable overexpression of Mcl-1 was intermittently tested by western blotting. A375 or SK-MEL-28 cells with stable overexpression of Mcl-1 were denoted as A375-Mcl-1+/+ or SK-MEL-28-Mcl-1+/+ respectively.

### Mcl-1 silencing

Silencing of Mcl-1 was achieved according to the protocol described by us previously [50]. Briefly, 0.3 × 10^6^ cells were plated in OPTI-MEM without antibiotics and transfected with Mcl-1 siRNA#1, Mcl-1 siRNA#2 or scrambled siRNA. Complexes were prepared by incubating 10nM siRNA with 8 μl siPORT transfection reagent in 200 μl OPTI-MEM media without serum or antibiotic for 20 minutes. These complexes were then added to the cells. Six hours after transfection, complexes were replaced with fresh medium.

### Tumor therapy

All the experiments involving animals were approved by the Institutional Animal Care and Use Committee. About 5–6 weeks old athymic nude mice (Charles River, Wilmington, MA, USA) were allowed to acclimatize for one week prior to the beginning of the experiments. Mice were injected subcutaneously with 3.5 × 10^6^ A375-VR cells. When the tumor volume reached to 150 mm^3^, mice were randomly segregated into several groups with 6–7 mice in each group. Vemurafenib, formulated as microprecipitated bulk powder (MBP) was suspended at a desired concentration in a vehicle containing 2% Klucel LF and adjusted to pH 4 with HCl, was given at a dose of 25 mg/kg twice a day through oral gavage. TW-37 in PBS/ethanol/Tween 80 was administered intraperitonially at a dose of 30 mg/kg thrice a week. Mcl-1 (0.2 nmol) or scrambled siRNA was administered twice a week directly into the tumors (intra-tumoral injection) as described by us previously [50]. Tumor measurements were taken thrice a week by vernier calipers and the volume was calculated by the formula V = length * (breadth)^2^/2 as previously described [51–53]. At the end of the experiment, animals were euthanized humanely and tumors were excised and fixed in formalin for immunohistochemistry or flash frozen in liquid nitrogen for western blot analysis as described previously by us [54].

### Immumnohistochemistry

Immunohistochemistry was performed according to the protocol previously described by us [55]. Expression of Mcl-1, cleaved caspase 3 and p-ERK1/2 in tumor samples were analyzed in paraffin sections obtained from five mice from each group. The sections were deparaffinized and rehydrated using decreasing concentrations of ethanol. Antigen retrieval process was carried out by boiling the sections in citrate buffer (pH 6) for 10 minutes. Endogenous peroxides were quenched by incubating the sections in 3% hydrogen peroxide solution. Sections were blocked using 6% goat serum for 30 minutes after which they were exposed to primary antibody overnight. Following the incubation, the expression was detected using Ultravision ONE detection reagent (Thermo Fisher, Houston, TX) according to the manufacturer's protocol. The sections were then counterstained with Mayer's hematoxylin and dehydrated using increasing concentrations of ethanol and xylene and observed under the microscope (Olympus). TUNEL assay was carried out according to the manufacturer's protocol.

### Patient samples

Patients with metastatic melanoma containing BRAF V600E mutation (confirmed by genotyping) were consented for tissue acquisition per IRB-approved protocol covered under DF/HCC protocol number 11–181. Tumor biopsies were performed pre-treatment (day 0) 10–14 days after treatment initiation (on treatment) and upon evidence of resistance to therapy (progression). Formalin-fixed tissue was analyzed to confirm that viable tumor was present via hematoxylin and eosin (H&E) staining.

### Statistical analysis

All the statistical analysis was performed using Prism 6.0 (Graph Pad software Inc., San Diego, CA, USA). *In vitro* data was plotted as mean ± S.D. of at least three independent experiments and *in vivo* data was plotted as mean ± S.E.M. Data was analyzed by Student's *t*-test or one way ANOVA followed by Tukey's *post-hoc* analysis for multiple comparisons. Differences were considered statistically significant at *P* < 0.05.

## SUPPLEMENTARY FIGURES AND TABLES


