# Supplementary material for: Overexpression of Mcl-1 confers resistance to BRAFV600E inhibitors alone and in combination with MEK1/2 inhibitors in melanoma
Source: Oncotarget. 2015 Oct 14;6(38):40535–56. doi: 10.18632/oncotarget.5755 (PMC4747351; doi:10.18632/oncotarget.5755)
Supplement: Supplementary file 1 [file oncotarget-06-40535-s001.pdf]

SUPPLEMENTARY FIGURES AND TABLES

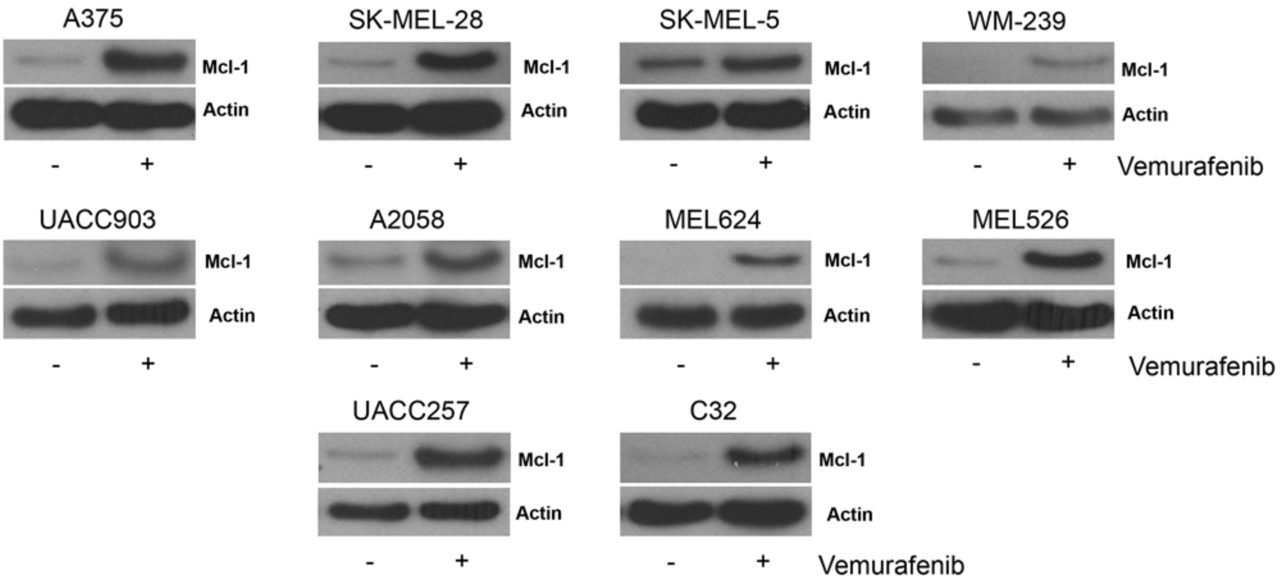

Supplementary Figure S1: Vemurafenib treatment induces Mcl-1 expression in melanoma cells. Ten BRAF mutant cell lines were treated with 0.4 μM vemurafenib for 72 hours. The protein was collected, subjected to western blotting and analyzed for Mcl-1 expression. Actin was used as a loading control. Each experiment was performed at least three times independently.

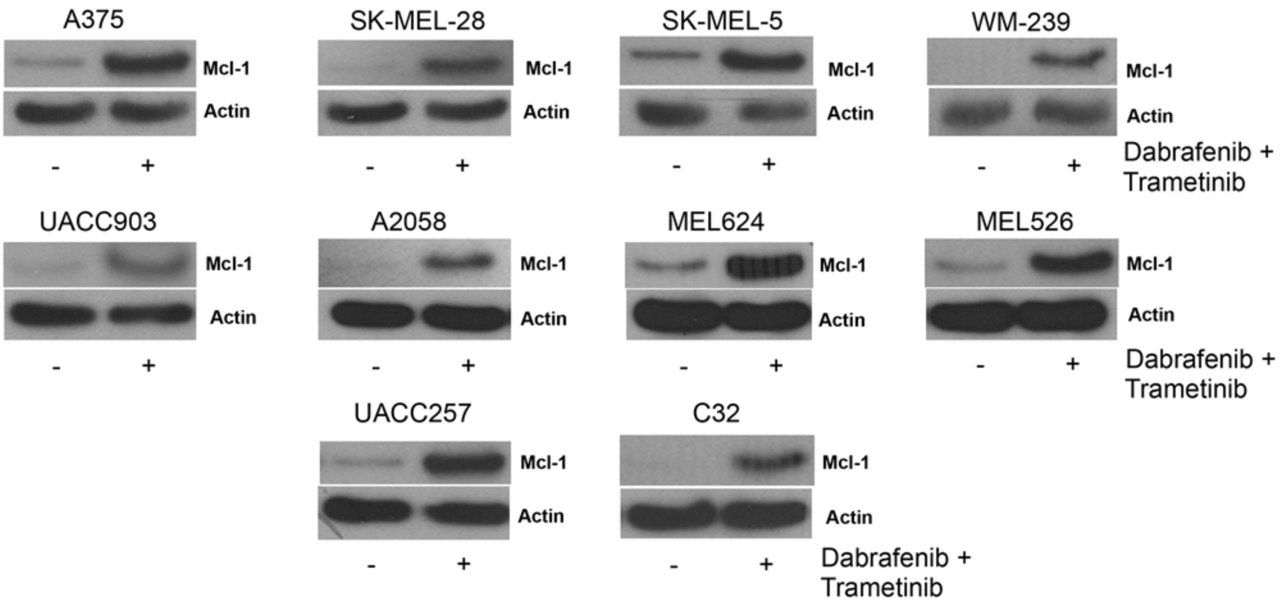

Supplementary Figure S2: Dabrafenib and Trametinib combination treatment induces Mcl-1 expression in melanoma cells. Ten BRAF mutant cell lines were treated with a combination of 10nM dabrafenib and 2nM trametinib for 72 hours. The protein was collected, subjected to western blotting and analyzed for Mcl-1 expression. Actin was used as a loading control. Each experiment was performed at least three times independently.

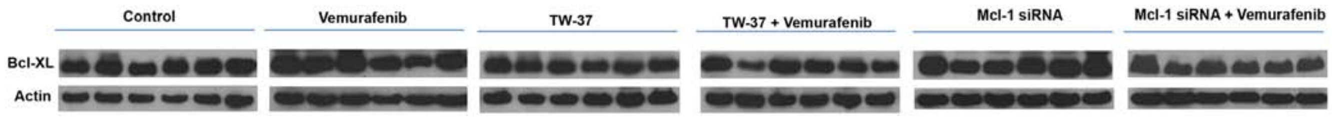

Supplementary Figure S3: TW-37 and Mcl-1 siRNA did not inhibit Bcl-XL. A375-VR cells were injected subcutaneously in female athymic nude mice. Once the tumor volume reach 150 mm^3, the mice were randomly divided into 5 groups ($n = 6$ in each group) and the treatment was started as described under 'Material and Method' section. After the termination of the experiment, tumor lysates from 6 mice were subjected to western blotting and analyzed for Bcl-XL. β actin was used as a loading control.

Supplementary Table S1: Sequences of siRNAs

Mcl-1 siRNA#1	AGCCUAGUAUGUCAAUAAAUUUAUUGACAUACUAGGCU
Mcl-1 siRNA#2	CCUAGUUUAUCACCAAUAAUUAUUGGUGAUAAACUAGG

Supplementary Table S2: Development of resistance to vemurafenib in A375 cells

Month	0	1	2	3	4	5	6	7	8	9	10	11	12
Vemurafenib (µM)	0	0.2	0.2	0.4	0.4	0.8	0.8	1	1	4	8	8	10
IC50 (µM)(A375)	0.1	0.215	0.285	0.41	0.45	0.485	0.56	0.85	0.97	1.25	1.84	2.6	3
Fold Resistance	1	2.15	2.85	4.1	4.5	4.85	5.6	8.5	9.7	12.5	18.4	26	30

Resistance to vemurafenib in A375 cells was developed by treating the cells with escalating concentrations of vemurafenib as shown in the table. Cells were exposed to two treatments of vemurafenib in a week for 72 hours each. Cytotoxicity of these cells was evaluated intermittently by sulforhodamine B assay.

Supplementary Table S3: Development of resistance to vemurafenib in SK-MEL-28 cells

Month	0	1	2	3	4	5	6	7	8	9	10	11	12
Vemurafenib (µM)	0	0.2	0.2	0.4	0.4	0.8	0.8	1	1	4	8	8	10
IC50 (µM)SK-MEL-28	0.075	0.158	0.162	0.264	0.315	0.61	0.673	0.735	0.754	0.824	1.236	1.864	3.3
FoldResistance	1	2.1	2.24	3.52	4.2	8.13	8.97	9.8	10.05	10.99	16.48	24.85	44

Resistance to vemurafenib in SK-MEL-28 cells was developed by treating the cells with escalating concentrations of vemurafenib as shown in the table. Cells were exposed to two treatments of vemurafenib in a week for 72 hours each. Cytotoxicity of these cells was evaluated intermittently by sulforhodamine B assay.
